# Hypercluster: a flexible tool for parallelized unsupervised clustering optimization

**DOI:** 10.1186/s12859-020-03774-1

**Published:** 2020-09-29

**Authors:** Lili Blumenberg, Kelly V. Ruggles

**Affiliations:** 1 Institute of Systems Genetics, New York University Grossman School of Medicine, New York, NY 10016 USA; 2 Department of Medicine, New York University Grossman School of Medicine, New York, NY 10016 USA

**Keywords:** Machine learning, Unsupervised clustering, Hyperparameter optimization, Scikit-learn, Python, SnakeMake

## Abstract

**Background:**

Unsupervised clustering is a common and exceptionally useful tool for large biological datasets. However, clustering requires upfront algorithm and hyperparameter selection, which can introduce bias into the final clustering labels. It is therefore advisable to obtain a range of clustering results from multiple models and hyperparameters, which can be cumbersome and slow.

**Results:**

We present hypercluster, a python package and SnakeMake pipeline for flexible and parallelized clustering evaluation and selection. Users can efficiently evaluate a huge range of clustering results from multiple models and hyperparameters to identify an optimal model.

**Conclusions:**

Hypercluster improves ease of use, robustness and reproducibility for unsupervised clustering application for high throughput biology. Hypercluster is available on pip and bioconda; installation, documentation and example workflows can be found at: https://github.com/ruggleslab/hypercluster.

## Background

Unsupervised clustering is ubiquitously used for the interpretation of ‘omics datasets [1–7]. Clustering is a particularly central challenge in the analysis of single-cell measurement data (e.g. single cell RNA-seq) due to its high dimensionality [8–10]. Clustering is also increasingly being used for disease subtype classification and risk stratification [11–19]. It is therefore essential that optimal clustering results are easily and robustly obtainable, without user-selected hyperparameters introducing bias and impeding rapid analysis.

Clustering is inherently under-defined [20–22]. The definition of “cluster” differs from problem to problem and the desired goal of the analysis [14], and therefore it is not possible to make a single algorithm or metric that can universally identify the “best” clusters [23]. Researchers therefore often compare results from multiple algorithms and hyperparameters [7, 24–28]. Typically, the effect of hyperparameter choice on the quality of clustering results cannot be described with a convex function, meaning that hyperparameters should be chosen through exhaustive grid search [29], a slow and cumbersome process. Software packages for automatic hyperparameter tuning and model selection for regression and classification exist, notably auto-sklearn from AutoML [30], and some groups have made excellent tools for distributing a single clustering calculation for huge datasets [31, 32], but to the best of our knowledge, there is no package for comparing several clustering algorithms and hyperparameters.

Here we present hypercluster, a python package and SnakeMake pipeline for rigorous, reproducible and parallelized clustering calculation and evaluation. This package allows users to compare multiple hyperparameters and algorithms, then easily visualize evaluation metrics for each result [33]. The SnakeMake pipeline allows parallelization, greatly reducing wall-clock time for users [34]. Hypercluster provides researchers with a flexible, parallelized, distributed and user-friendly method for clustering algorithm selection and hyper-parameter tuning.

## Implementation

### Requirements

**Fig. 1:**
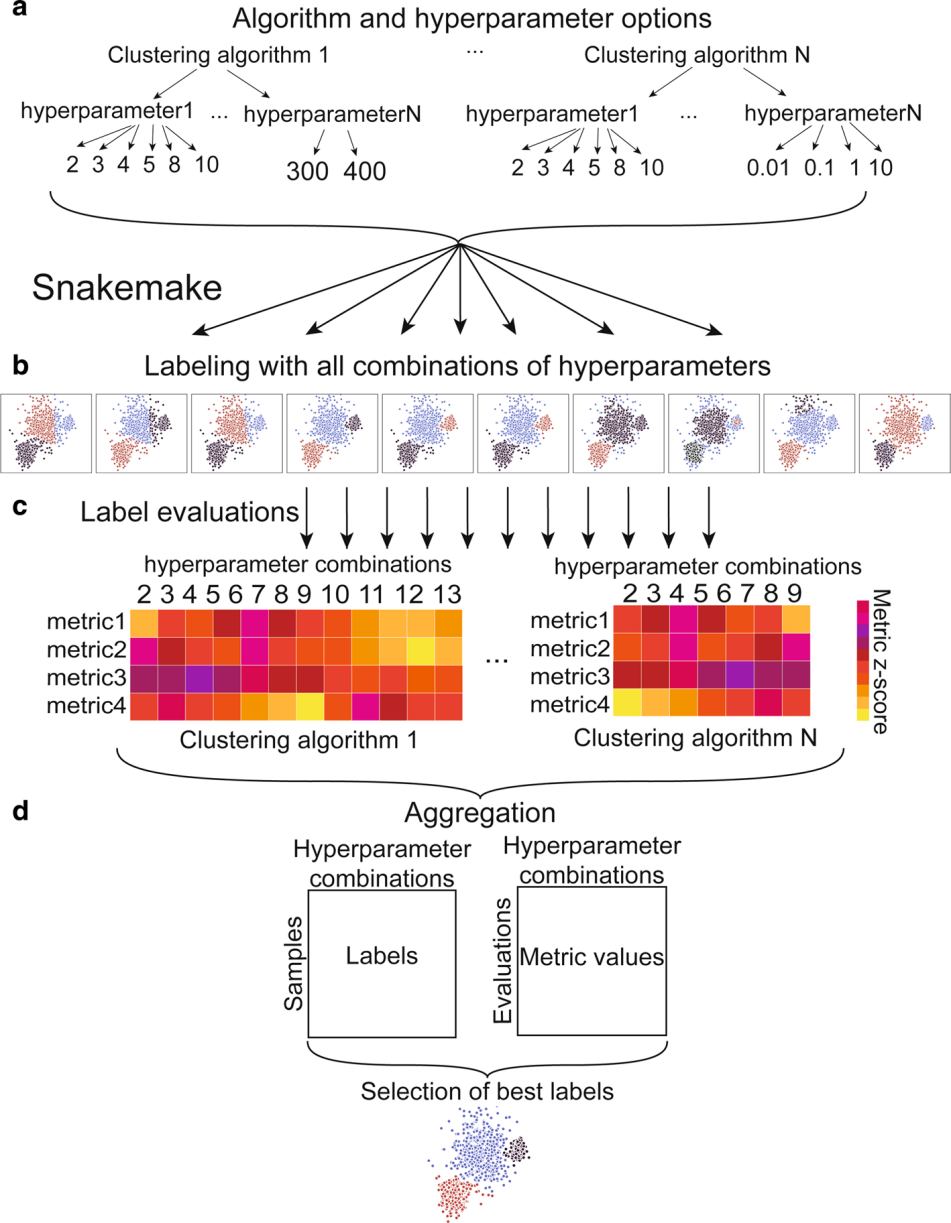
Hypercluster workflow schematic. **a** Clustering algorithms and their respective hyperparameters are user-specified. Hypercluster then uses those combinations to create exhaustive configurations, and if selected a random subset is chosen. **b** Snakemake is then used to distribute each clustering calculation into different jobs. **c** Each set of clustering labels is then evaluated in a separate job by a user-specified list of metrics. **d** All clustering results and evaluation results are aggregated into tables. Best labels can also be chosen by a user-specified metric.

The hypercluster package uses scikit-learn [35], python-igraph [36], leidenalg [37] and louvain-igraph [38] to assign cluster labels and uses scikit-learn and custom metrics to compare clustering algorithms and hyperparameters to find optimal clusters for any given input data (Fig. 1). Hypercluster requires python3, pandas [39], numpy [40], scipy [41], matplotlib [42], seaborn [43], scikit-learn [35], python-igraph [36], leidenalg [37], louvain-igraph [38] and SnakeMake [34].

### General workflow and examples

Hypercluster can be run independently of SnakeMake, as a standalone python package. Input and output structure, as well as example workflows on a breast cancer RNA-seq data set [43] and scRNA-seq [45] can be found at https://github.com/ruggleslab/hypercluster/tree/master/examples. Briefly, the workflow starts with instantiating an AutoClusterer (for a single algorithm) or MultiAutoClusterer (for multiple algorithms) object with default or user-defined hyperparameters (Fig. 1a). To run through hyperparameters for a dataset, users simply provide a pandas DataFrame to the “fit'' method on either object (Fig. 1b). Users evaluate the labeling results with a variety of metrics by running the “evaluate” method (Fig. 1c). Clustering labels and evaluations are then aggregated into convenient tables (Fig. 1d), which can be visualized with built in functions (e.g. Additional file 1: Fig. S1, Additional file 2: Fig. S2).

### Configuring the SnakeMake pipeline

The SnakeMake pipeline allows users to parallelize clustering calculations on multiple threads on a single computer, multiple compute nodes on a high performance cluster or in a cloud cluster [34]. The pipeline is configured through a config.yml file (Table 1), which contains user-specified input and output directories and files (Table 1, lines 1–3, 5–7) and the hyperparameter search space (Fig. 1a, Table 1, line 18). This file contains predefined defaults for the search space that allow the pipeline to be used “out of the box.” Further, users can specify whether to use exhaustive grid search or random search; if random search is selected, probability weights for each hyperparameter can be chosen (Table 1, line 9). The pipeline then schedules each clustering calculation and evaluation as a separate job (Fig. 1b). Users can specify which evaluation metrics to apply (Fig. 1c, Table 1, line 10) and add keyword arguments to tune several steps in the process (Table 1, lines 4, 8–9, 11–16). Clustering and evaluation results are then aggregated into final tables (Fig. 1d). Users can reference the documentation and examples for more information.

**Table 1:** Parameters in SnakeMake configuration file

config.yml parameter	Explanation	Example
1 input_data_folder	Path to folder in which input data can be found	/input_data
2 input_data_files	List of prefixes of data files	['input_data1’, 'input_data2’]
3 gold_standard_file	File name of gold_standard_file, must be in input_data_folder	{'input_data': 'gold_standard_file.txt'}
4 read_csv_kwargs	pandas.read_csv keyword arguments for input data	{'test_input': {'index_col':[0]}}
5 output_folder	Path to folder into which results should be written	/results
6 intermediates_folder	Name of subfolder to put intermediate results	clustering_intermediates
7 clustering_results	Name of subfolder to put aggregated results	clustering
8 clusterer_kwargs	Additional arguments to pass to clusterers	KMeans: {'random_state':8}}
9 generate_parameters_addtl_kwargs	Additonal keyword arguments for the hypercluster.AutoClusterer class	{‘KMeans’: {'random_search': true)
10 evaluations	Names of evaluation metrics to use	['silhouette_score', 'number_clustered']
11 eval_kwargs	Additional kwargs per evaluation metric function	{'silhouette_score': {'random_state': 8}}
12 metric_to_choose_best	Which metric to maximize to choose the labels	silhouette_score
13 metric_to_compare_labels	Which metric to use to compare label results to each other	adjusted_rand_score
14 compare_samples	Whether to made a table and figure with counts of how often each two samples are in the same cluster	"true"
15 output_kwargs	pandas.to_csv and pandas.read_csv keyword arguments for output tables	{'evaluations': {'index_col':[0]}, 'labels': {'index_col':[0]}}
16 heatmap_kwargs	Arguments for seaborn.heatmap for pairwise visualizations	{'vmin':-2, 'vmax':2}
17 optimization_parameters	Which algorithms and corresponding hyperparameters to try	{'KMeans': {'n_clusters': [5, 6, 7] }}

As input, users provide a data table with samples to be clustered as rows and features as columns. Users can then simply run “snakemake -s hypercluster.smk -configfile config.yml” in the command line, with any additional SnakeMake flags appropriate for their system. Applying the same configuration to new files or testing new algorithms on old data simply requires editing the inputs in the config.yml file and rerunning the SnakeMake command.

### Extending hypercluster

Currently, hypercluster can perform any clustering algorithm and calculate any evaluation available in scikit-learn [35, 46], as well as non-negative matrix factorization (NMF) [47], Louvain [38] and Leiden [37] clustering. Additional clustering classes and evaluation metric functions can be added by users in the additional_clusterer.py and additional_metrics.py files, respectively, if written to accommodate the same input, outputs and methods (see additional_clusterers.py and additional_metrics.py for examples).

### Outputs

For each set of labels, hypercluster generates a file with sample labels and a file containing evaluations of those labels. It also outputs aggregated tables of all labels and evaluations. Hypercluster can also generate several helpful visualizations, including a heatmap showing the evaluation metrics for each set of hyperparameters (Fig. 1c) and a table and heatmap of pairwise comparisons of labeling similarities with a user-specified metric (Additional file 1: Fig. S1). This visualization is particularly useful for finding labels that are robust to differences in hyperparameters. It can also optionally output a table and heatmap showing how often each pair of samples were assigned the same cluster (Additional file 2: Fig. S2). Other useful custom visualizations that are simple for users to create due to the aggregated clustering results are available in our examples (https://github.com/ruggleslab/hypercluster/tree/dev/examples).

## Conclusions

Hypercluster allows comprehensive evaluation of multiple hyperparameters and clustering algorithms simultaneously, reducing the allure of biased or arbitrary parameter selection. It also aids computational biologists who are testing and benchmarking new clustering algorithms, evaluation metrics and pre- or post-processing steps [10]. Future iterations of hypercluster could include further cutting-edge clustering techniques, including those designed for larger data sets [31, 32] or account for multiple types of data [48]. Hypercluster streamlines comparative unsupervised clustering, allowing the prioritization of both convenience and rigor.

## Availability and requirements

- Project Name: Hypercluster.
- Project homepage: https://github.com/ruggleslab/hypercluster/.
- Operating system: Platform independent.
- Programming Language: Python.
- Other requirements: Hypercluster runs with the following versions or higher: python 3.7, pandas 0.24.2, numpy 1.16.4, scipy 1.2.1, matplotlib 3.1.0, seaborn 0.9.0, scikit-learn 0.22.0, hdbscan 0.8.24, snakemake 5.8.2, python-igraph 0.7.1, leidenalg 0.7.0, louvain 0.6.1
- License: MIT license, open for use by academic and non-academic users.
- Any restrictions to use by non-academics: Not applicable.

## Supplementary information

**Additional file 1**. **Figure S1**: Pairwise label comparisons. Automatically generated heatmap showing pairwise comparison of labeling automatically generated using hypercluster of breast cancer samples. Colors represent adjusted rand index between labels.

**Additional file 1**. **Figure S2**: Pairwise sample comparisons. Automatically generated pairwise comparison of breast cancer samples. Color indicates the number of times two samples were assigned the same cluster.

## Data Availability

Source code, as well as example vignettes, is available at https://github.com/ruggleslab/hypercluster.

## Abbreviations

NMF: Non-negative matrix factorization
scRNA-Seq: Single cell RNA-seq
TCGA: The Cancer Genome Atlas
