# Supplementary figures and images for: Hypercluster: a flexible tool for parallelized unsupervised clustering optimization
Source: BMC Bioinformatics. 2020 Sep 29;21:428. doi: 10.1186/s12859-020-03774-1 (PMC7525959; doi:10.1186/s12859-020-03774-1)

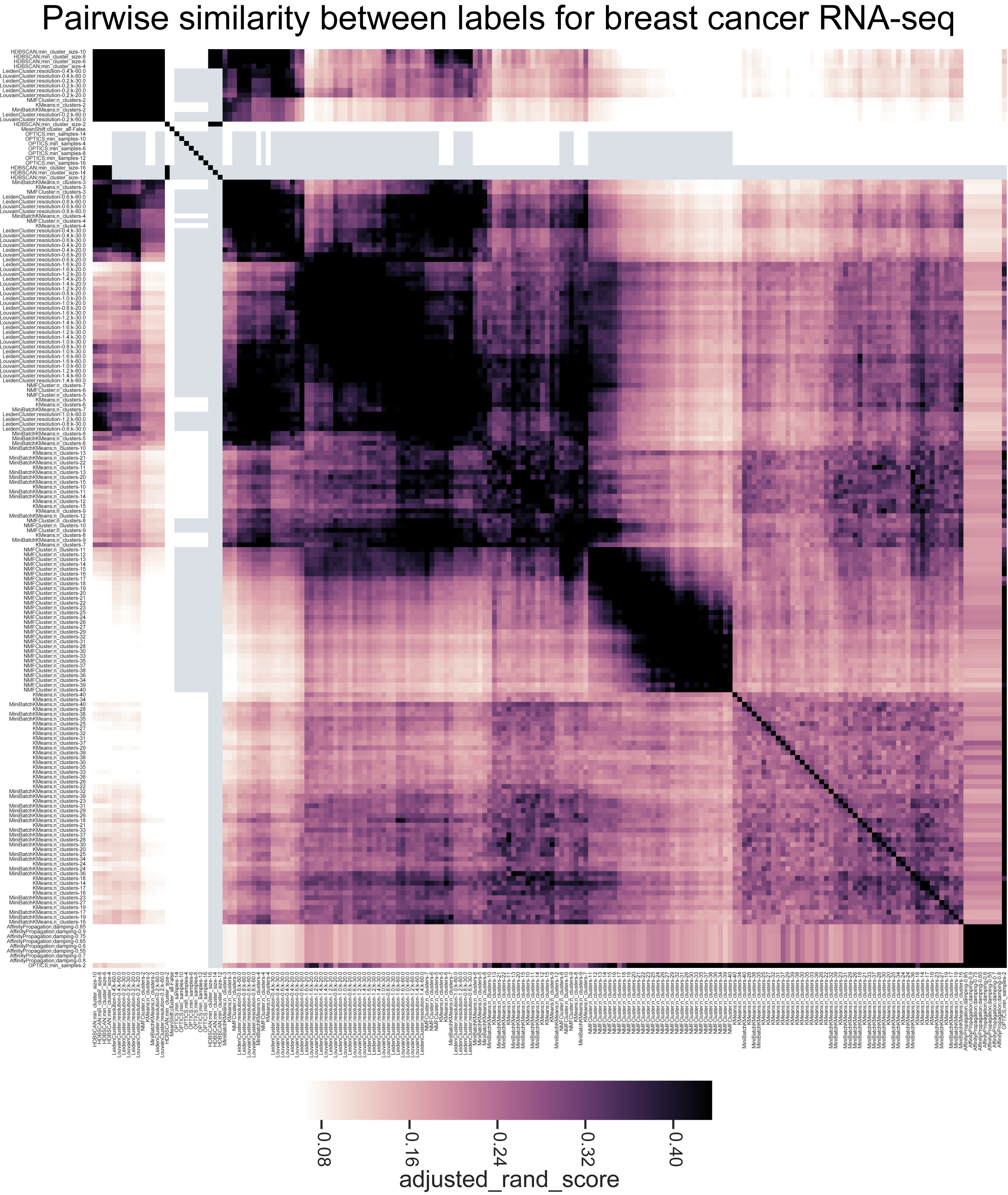

Supplement: Supplementary file 1 — Additional file 1. Figure S1: Pairwise label comparisons. Automatically generated heatmap showing pairwise comparison of labeling automatically generated using hypercluster of breast cancer samples. Colors represent adjusted rand index between labels. [file 12859_2020_3774_MOESM1_ESM.tif]

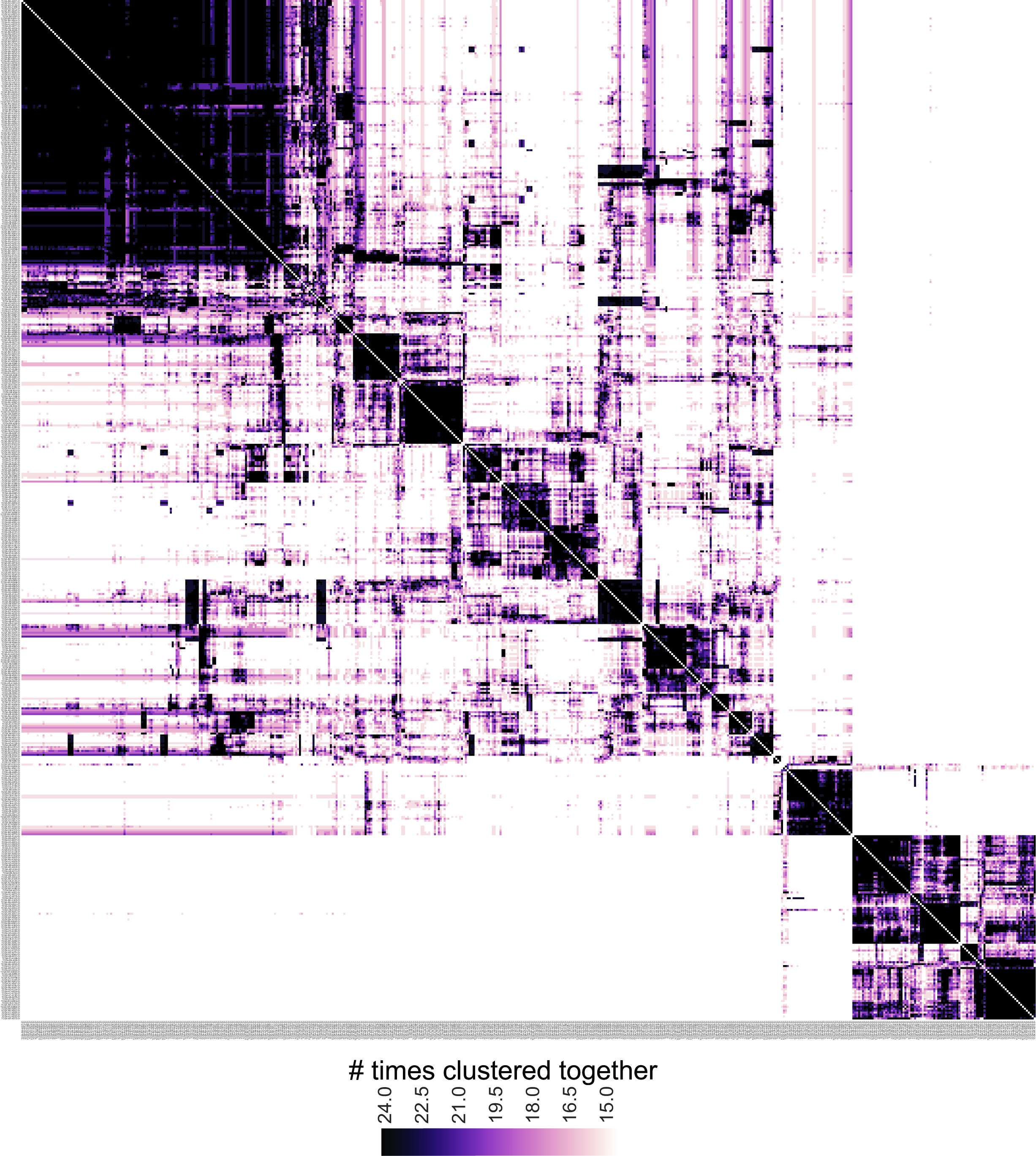

Supplement: Supplementary file 2 — Additional file 1. Figure S2: Pairwise sample comparisons. Automatically generated pairwise comparison of breast cancer samples. Color indicates the number of times two samples were assigned the same cluster. [file 12859_2020_3774_MOESM2_ESM.tif]
